# Functionally-selective inhibition of threshold sodium currents and excitability in dorsal root ganglion neurons by cannabinol

**DOI:** 10.1038/s42003-024-05781-x

**Published:** 2024-01-23

**Authors:** Mohammad-Reza Ghovanloo, Philip R. Effraim, Sidharth Tyagi, Peng Zhao, Sulayman D. Dib-Hajj, Stephen G. Waxman

**Affiliations:** 1 Department of Neurology, Yale University School of Medicine, New Haven, CT USA; 2 Center for Neuroscience & Regeneration Research, Yale University, West Haven, CT USA; 3 Neuro-Rehabilitation Research Center, Veterans Affairs Connecticut Healthcare System, West Haven, CT USA; 4 Department of Anesthesiology, Yale University School of Medicine, New Haven, CT USA; 5 Medical Scientist Training Program, Yale University School of Medicine, New Haven, CT USA

**Keywords:** Somatic system, Neuropathic pain

## Abstract

Cannabinol (CBN), an incompletely understood metabolite for ∆9-tetrahydrocannabinol, has been suggested as an analgesic. CBN interacts with endocannabinoid (CB) receptors, but is also reported to interact with non-CB targets, including various ion channels. We assessed CBN effects on voltage-dependent sodium (Nav) channels expressed heterologously and in native dorsal root ganglion (DRG) neurons. Our results indicate that CBN is a functionally-selective, but structurally-non-selective Nav current inhibitor. CBN’s main effect is on slow inactivation. CBN slows recovery from slow-inactivated states, and hyperpolarizes steady-state inactivation, as channels enter deeper and slower inactivated states. Multielectrode array recordings indicate that CBN attenuates DRG neuron excitability. Voltage- and current-clamp analysis of freshly isolated DRG neurons via our automated patch-clamp platform confirmed these findings. The inhibitory effects of CBN on Nav currents and on DRG neuron excitability add a new dimension to its actions and suggest that this cannabinoid may be useful for neuropathic pain.

## Abstract (web summary)

Cannabinol (CBN) is a promising compound that is suggested as an analgesic. Using electrophysiological techniques, this study determined that CBN inhibits the sodium currents associated with pain signaling, with potential application for neuropathic pain.

## Introduction

Cannabis contains many biologically active compounds including phytocannabinoids^1,2^. Despite recent efforts, there are many minor cannabinoids that remain understudied. Cannabinol (CBN) is a minor and understudied cannabinoid, which is synthesized as a result of oxidation of ∆9-tetrahydrocannabinol (THC) which causes aromatization at the level of methyl moieties. Given its direct chemical relationship with THC, CBN shares some pharmacological features with THC^3^.

Like cannabidiol (CBD) and cannabigerol (CBG), CBN has been suggested as an anti-inflammatory and analgesic agent^4,5^. Previous studies have reported that CBN (dosed at 1 mg/ml) can mitigate myofascial pain in rats^6^. CBN is also being investigated for ophthalmologic disorders including glaucoma^7,8^. Finally, CBN has been suggested to possess antioxidant and antibacterial effects^9^.

The most important receptors in the endocannabinoid system are CB1 and CB2^10^. The activity of some cannabinoids at these receptors is linked with euphoric psycho-activity. THC was first discovered to interact with these receptors^11^. Recently, other non-psychotropic phytocannabinoids, including CBD and CBG, were shown to interact with several other targets^1,12^, including multiple receptors, ion channels, and the cell membrane itself^4^. Like CBD and CBG^4^, CBN modulates several transient receptor potential (TRP) channels^13^. Although CBN is an agonist at both CB receptors, it has been classified as a non-psychoactive cannabinoid that possess sedative-like properties^3^.

Among the potential therapeutic applications for CBN, its analgesic effects are particularly interesting. Because CBN shares analgesic features and diversity of known molecular targets with both CBD and CBG, we sought to determine if CBN also modulates voltage-gated sodium (Nav) channels, which have a well-stablished role in pain physiology and are implicated as vital to the CB-independent cannabinoid pathway^4,14^. Nav1.7 is regarded as the major Nav isoform that sets the gain for nociception in DRG neurons^15–18^ and is being explored as a target for analgesic drug development. Whether CBN, like CBD and CBG, acts on Nav1.7 (and others) and attenuate neuronal firing is not known.

Previous studies thoroughly described the interactions between Nav channels and CBD/CBG. CBD was shown to directly block Nav channels as well as indirectly inhibit their activity through modulating bio-membrane stiffness; CBG was shown to inhibit maximal sodium conductance (*G*_max_) more potently than stabilizing inactivation^4^. Given the similarities in the structures and other features of CBD/CBG with CBN, we used earlier observations of CBD/CBG to guide our investigation of CBN in this study. Here, using multiple electrophysiological techniques, we show that CBN is a functionally selective inhibitor of Nav currents and of DRG neuron excitability. Our results suggest that CBN may be a promising compound for various types of pain, including neuropathic pain.

## Results

### CBN is a state-dependent inhibitor of Nav1.7 sodium currents

**Fig. 1:**
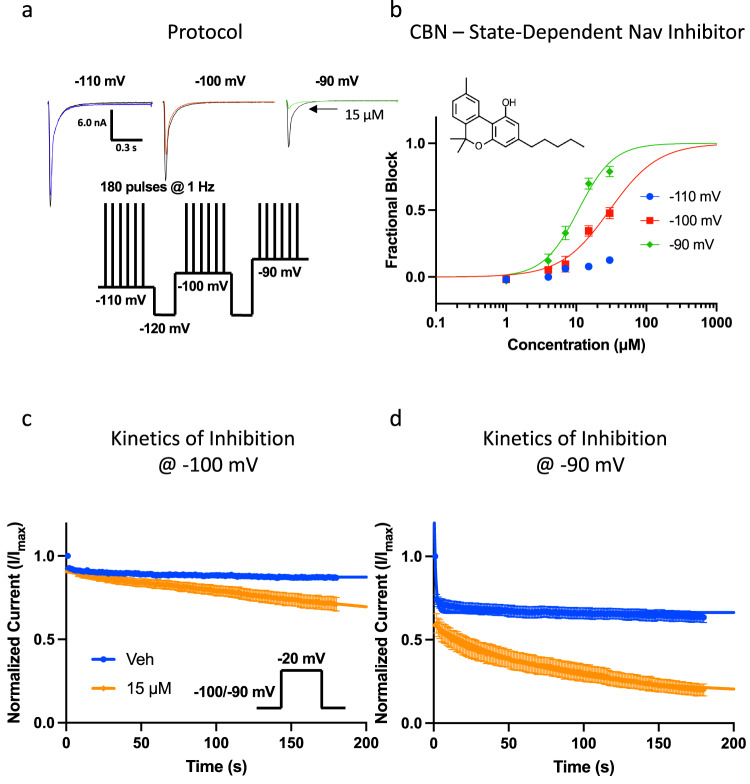
State-dependence of CBN as a Nav channel inhibitor. **a** Pulse protocol showing 180 pulses run at 1 Hz at each holding-potential and representative current traces. The arrow indicating 15 µM is pointing to smaller superimposed current trace which indicates inhibition imparted by CBN. The leftmost trace in blue shows that 15 µM does not comparatively inhibit as much of the Nav1.7 current as the orange trace (middle) or the green trace (rightmost). Each trace indicates data associated with one of the three holding-potentials that are indicated. **b** CBN potency at varying holding-potentials at pulse 180 (3 min) in Nav1.7 (IC_50_ (µM): −100 mV = 29.9 ± 3.2, −90 mV = 10.7 ± 0.8; Hill coefficient: −110 mV = −100 mV = 1.3 ± 0.2, −90 mV = 1.7 ± 0.2; *n* = 12–26). Structure of CBN is shown at the top left. **c** Kinetics of inhibition of Nav1.7 at −100 mV, **d** and −90 mV holding-potentials (Mean tau (s): −100 mV Veh = 6.3 ± 0.9, −100 mV CBN = 51.4 ± 4.0, −90 mV Veh = 1.1 ± 0.4; −90 mV CBN = 76.2 ± 11.5, *n* = 8–15).

Our primary objective in this study was to determine the concentration-response of CBN on voltage-dependent Nav currents, and also determine if channels are inhibited in a state-dependent manner. We used patch-clamp to record macroscopic sodium currents in HEK293 cells that stably expressed human Nav1.7. We used a protocol to study state-dependent inhibition over a range of holding-potentials with varying channel inactivation^19^. First, the channels were held at a holding-potential of −110 mV, where Nav1.7 is almost fully in the resting state, then we pulsed the channels 180 times at 1 Hz for the drug to reach equilibrium with the channels. Next, we depolarized the holding-potential by 10 mV two more times and repeated the 3-minute pulse train at each voltage of −100 and −90 mV (Fig. 1a). To construct the concentration-response curves, each of the cells were perfused with one concentration of CBN, then the normalized inhibition at each concentration was pooled and fitted to the Hill-Langmuir equation to obtain the IC_50_ and Hill-slope. We show representative current traces in Fig. 1a. We also show the fractional block of sodium currents from the final pulse from each holding-potential in Fig. 1b. We found that from a −110-mV holding-potential, when Nav1.7 is almost at full rest, CBN barely inhibited the sodium currents, even at the higher concentrations of 15–30 µM (Fig. 1b). However, as the holding-potential was depolarized, CBN began inhibiting the sodium currents, with the apparent potency increasing along with the changing holding-potential. At these latter holding-potentials CBN inhibited Nav1.7 with IC_50_ of 10.7 (at −90 mV) to 29.9 µM (at −100 mV), and Hill-slopes ranging from 1.3–1.7, respectively. Steep Hill-slopes of >1 suggest the presence of multiple interactions culminating in inhibition. Thus, our results suggest that there are multiple interactions contribute to CBN’s inhibition of Nav1.7, which could be indictive of allosteric activity at the bio-membrane level. This could be explained by CBN loading up into the membrane, contributing to a steep Hill-slope. These findings are similar to our previous observations in CBD and CBG^1,4,20,21^. A recent structural study discovered a new binding site for CBD adjacent to the IFM motif of Nav1.7^22^. It is possible that CBN might also interact with this site.

Next, we measured the kinetics of Nav1.7 inhibition by CBN. This was done via measuring the maximal sodium current’s amplitude over the time course of 3 minutes, while we applied pulses to −20 mV from the set holding-potentials of −100 and −90 mV. The kinetics were only measured at these potentials, as these were the only potentials at which substantial inhibition was observed. The observed equilibrium rate of inhibition (observed time constant, **τ**_obs_) was measured by fitting a single exponential decay to the current inhibition. Inhibition levels were normalized and plotted against time elapsed after addition of Veh/CBN (15 μM) set at time = 0 (Fig. 1c, d). We found that CBN’s rate of Nav1.7 inhibition becomes faster as the holding-potential becomes depolarized. This voltage-dependent increase in inhibition kinetics is consistent with the IC_50_ curves in Fig. 1b. These findings further suggest that CBN is a state-dependent Nav inhibitor.

### CBN prevents the opening of Nav1.7 channels, but does not alter their activation voltage-dependence

**Fig. 2:**
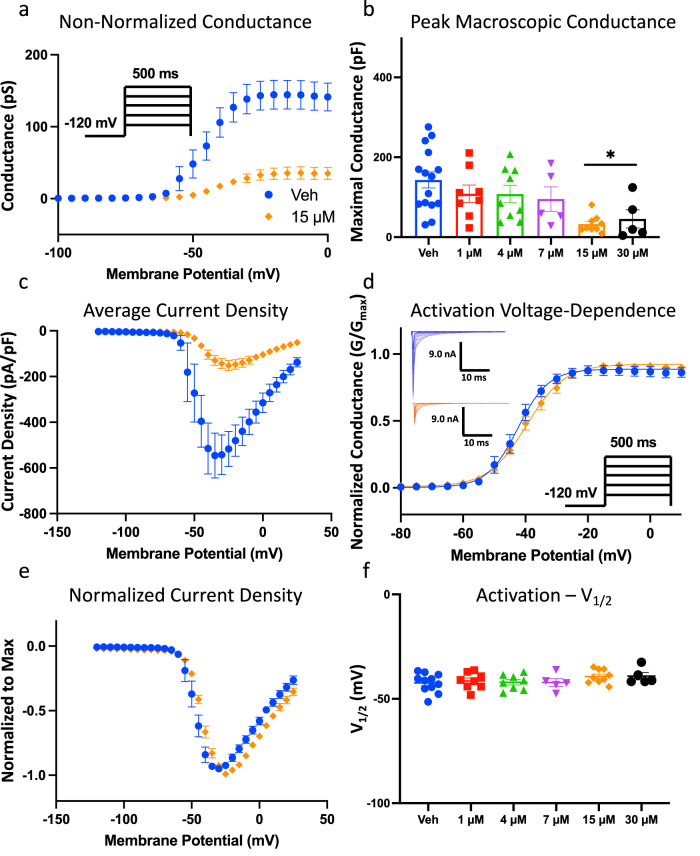
CBN does not affect activation, but it inhibits conductance in Nav1.7. **a** Conductance difference in Nav1.7 in vehicle and 15 µM CBN as a function of membrane potential. The holding-potential was −120 mV. The channels were held at −120 mV, followed by a series of step pulses at ∆5 mV, with each step being 500 ms long (see inset protocol inside of the panel). **b** Quantification of apparent peak macroscopic conductance at −25 mV across different CBN concentrations. Data shown as means ± SEM (*n* = 5–15). **c** Mean current density of hNav1.7 in vehicle and 15 µM CBN as a function of membrane potential. **d** Voltage-dependence of activation as normalized conductance plotted against membrane potential (Vehicle: *V*_1/2_ = −42.4 ± 1.4 mV, Slope = 3.9 ± 0.4, *n* = 11; CBN: *V*_1/2_ = −39.3 ± 1.1 mV, Slope = 5.9 ± 0.5, *n* = 9). **e** Normalized current density displaying unaltered activation. **f** Midpoints of activation across CBN concentrations. Data shown as means ± SEM (*n* = 5–15).

In contrast to the protocol that we used in Fig. 1a, where a series of repeating square pulses were used to measure intrinsic drug-induced inhibition of peak current amplitude, we carried out a more physiologically-relevant investigation of drug effect on apparent channel conductance, obtained across membrane potentials which would be more reflective of slight variations in potential in a cell membrane at resting conditions as well as post local depolarizations. To make these investigations we sought to determine whether CBN alters activation of Nav1.7 by measuring peak channel conductance at membrane potentials between −120 and +25 mV (from a −120-mV holding-potential between steps) with steps that were ∆5 mV apart. Figure 2a shows the effects of 15 µM CBN on peak conductance as obtained from 500 ms pulses as a function of membrane potential (As the steps were 500 milliseconds long, the measured peak from each step reflects a combination of *G*_max_ and inactivation, thus these numbers reflect the apparent *G*_max_ which we refer to as *G*_max_^*^). We found that ~80% of the sodium conductance was inhibited at 15 µM CBN. We also plotted the *G*_max_^*^ in cells perfused with 1–30 µM CBN at −25 mV (test-pulse at which maximal current was elicited). The data shown in Fig. 2b are a re-plotting of the maximal *G*_max_^*^ interval from Fig. 2a, which was to −25 mV. This re-plotting illustrates the CBN effect on the apparent Nav conductance at the potential which elicited the biggest *G*_max_^*^. The results indicate that CBN concentration-dependently decreases the Nav1.7 *G*_max_^*^ (without impacting *G*_max_, as indicated in Fig. 1) (Fig. 2b, Table S1). This decrease became statistically significant relative to vehicle at 15 µM (*p* < 0.05).

Next, we show a plot of sodium current density (expressed as peak I_Na_ divided by membrane capacitance; pA/pF) as a function of membrane potential. The CBN-mediated inhibition in current density was consistent with the effects on *G*_max_^*^, and showed a ~ 80% reduction in magnitude at 15 µM (Fig. 2c). In Fig. 2d, we show the normalized conductance plotted against membrane potential. These results demonstrate that none of the CBN concentrations induced significant changes in the midpoint (V_1/2_) of activation of the available Nav channels (p > 0.05) (Fig. 2d–f). Thus, exposure to CBN concentration-dependently prevents a sub-population of Nav1.7 channels from conducting; however, these exposures do not alter the voltage-dependence of activation in the remaining fraction of channels that are still available to activate. This effect is similar to that reported for CBD and CBG^4,20,21^. Collectively, the data presented in Fig. 2 indicate that the presence of CBN at >15 µM prevents opening of a majority of channels (or sodium ionic current conduction through Nav channels) but does not alter the voltage-dependence of activation. These results suggest that the sub-population of channels that do not conduct (i.e., *G*_max_^*^ inhibited) is likely inhibited by stabilization of channel inactivation.

### CBN hyperpolarizes inactivation; does not affect open-state inactivation

To further examine the effect of CBN on inactivation we then measured the voltage-dependence of steady-state inactivation (SSI) from a pre-pulse duration of 500 ms. Generally, durations in the range of less than a few hundred milliseconds are considered more implicit for fast-inactivation than for slow inactivation^23^. 500 ms is considered to trigger an intermediate amount of inactivation^21,24^. Using longer pre-pulses to measure inactivation is more physiological for Nav1.7, which is predominantly present in cells (e.g., DRG and trigeminal neurons) where the resting membrane potential (RMP) is significantly depolarized compared to channel availability *V*_1/2_ (RMP = ~−60 mV)^25,26^.

**Fig. 3:**
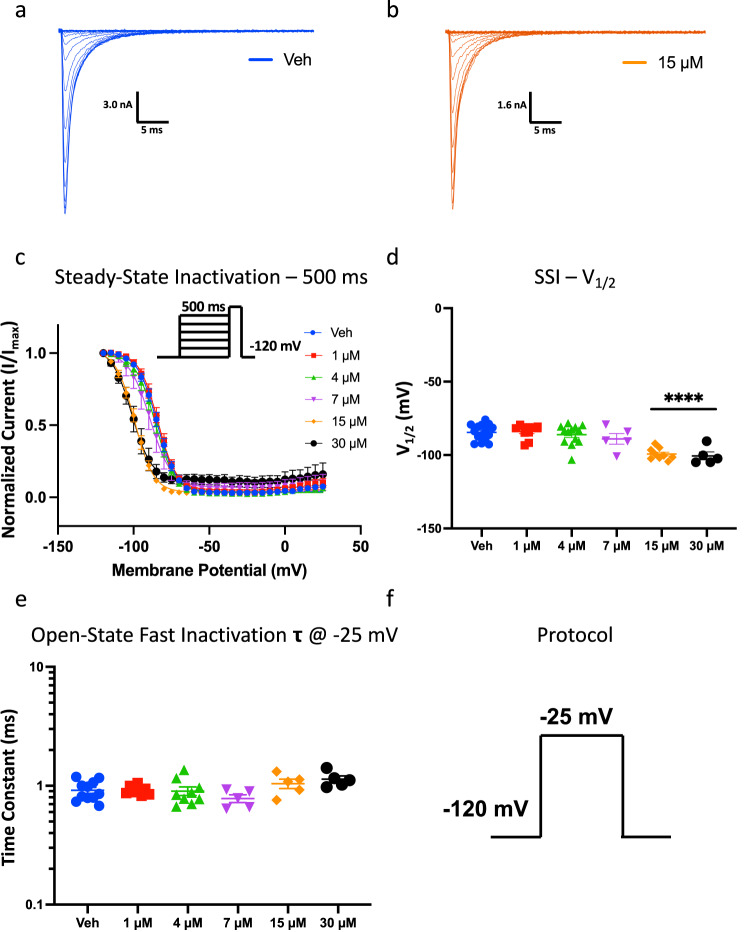
CBN hyperpolarizes 500 ms inactivation probability curve in Nav1.7, but it does not alter open-state fast-inactivation. **a**, **b** Representative macroscopic current traces of Veh and CBN (15 µM). **c** Voltage-dependence of 500 ms inactivation as normalized current plotted against membrane potential fit with single Boltzmann. **d** Quantification of SSI midpoints (in mV). Data shown as means ± SEM (*n* = 5–15). **** Indicates *p* < 0.0001, 15 and 30 µM compared to vehicle. **e** Open-state fast-inactivation time constants. Data shown as means ± SEM (*n* = 5–11). **f** The protocol that was used for **e**.

We show a normalization of current amplitudes at the test-pulse as a function of 500 ms pre-pulse voltages (Fig. 3a–c). We found that there is a concentration-dependent shift in the inactivation curves and this shift became statistically significant relative to vehicle at 15 µM CBN (Fig. 3d, Table S1). The current amplitude at our test-pulse was inhibited by ~80% at 15 µM. In contrast to activation where the voltage-dependence remained unchanged, the voltage-dependence of SSI of the uninhibited current was hyperpolarized, by a magnitude of ~15 mV (*p* < 0.0001). This indicates that CBN increased the likelihood of Nav1.7 channels to inactivate over the time course of the 500 ms pre-pulse, in the population of channels that were not inhibited from opening. This further suggests that CBN stabilizes the inactivated states of Nav1.7. CBN’s overall effect of hyperpolarizing inactivation is similar to that reported for CBD and CBG^1,4,20,21,27^.

We next measured the time constant associated with open-state inactivation at −25 mV. This type of inactivation is known as true fast-inactivation^28^. We measured the kinetics fitting the inactivating traces at −25 mV using an exponential function. We found that there were no significant differences at any of the CBN concentrations (1–30 µM) compared to vehicle (p > 0.05) (Fig. 3e, f). This suggests that CBN does not alter true fast-inactivation (fast-inactivation kinetics) and does not interact with the open-state, which is similar to CBD and CBG^1,4,20,21,27^.

### CBN slows the kinetics of Nav1.7 recovery from slow inactivation

**Fig. 4:**
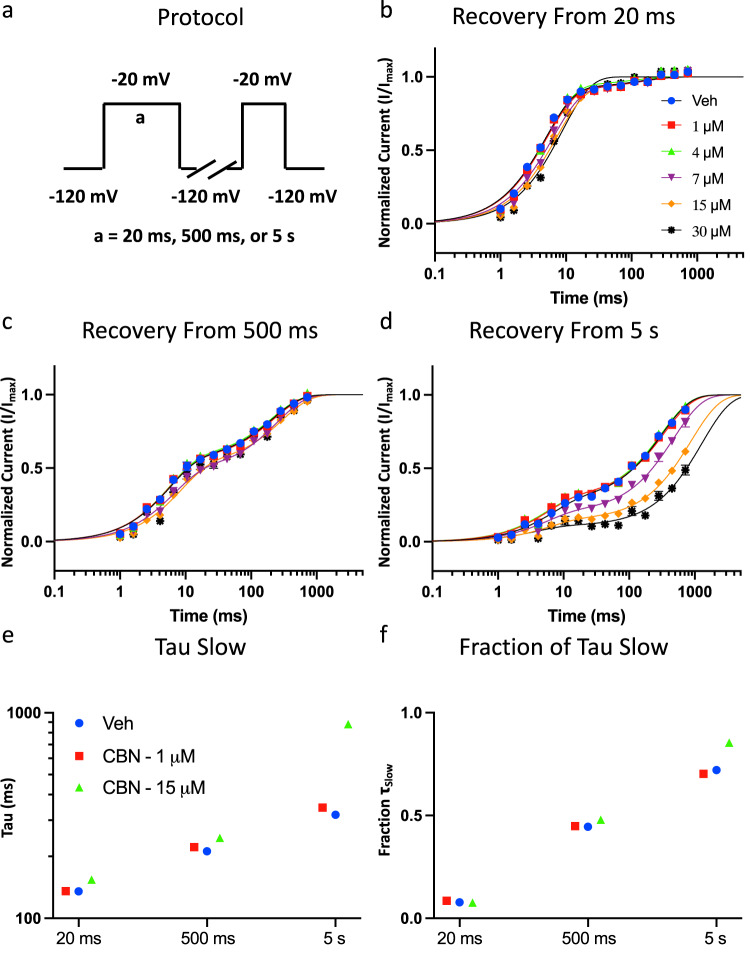
CBN slows recovery from slow inactivation in Nav1.7. **a** Shows the protocol that was used to measure CBG effect on channel recovery from duration pre-pulses. **b** Recovery from inactivation in the presence of 0 (Veh)−30 µM CBN, from 20 ms, **c** 500 ms, and **d** 5 s. Symbols for data shown in panels b–d are the same. Data shown as means ± SEM (*n* = 10–25 for 20 ms, 9–26 for 500 ms, and 5–23 for 5 s). **e**, **f** The slow components of recovery from inactivation in vehicle and CBN at 20 ms, 500 ms, and 5 s are shown on *Y* axis (**e**), and the fraction of slow to fast component of recovery from inactivation is shown on the *Y* axis (**f**). Symbols for data shown in panels e-f are the same.

To investigate the time-dependence and extent of CBN’s stabilization of inactivation, we measured the kinetics of recovery from inactivation of Nav1.7 at various CBN concentrations (1–30 µM). This was done after depolarizing pre-pulse durations of 20 ms, 500 ms, and 5 s corresponding to fast, intermediate, or slow inactivated states, from a holding-potential of −120 mV. The recovery from inactivation was measured by holding the channels at −120 mV to ensure that the channels were fully available, followed by pulsing the channels to −20 mV at one of the noted durations and allowed different time intervals at −120 mV to measure recovery as a function of time (Fig. 4a). The average normalized recovery following the pre-pulse in vehicle and different CBN concentrations are plotted and fitted with a biexponential function (Fig. 4b–d).

Our results suggested that if the channels are fast inactivated (20 ms), CBN does not affect recovery kinetics at any of the measured concentrations (Fig. 4b). This effect is similar even at the intermediate interval (500 ms) (Fig. 4c). However, during slow inactivation, which was triggered after 5 seconds of inactivation accumulation, CBN concentration-dependently slowed the recovery of inactivation (Fig. 4d). The slowed kinetics became more pronounced at 7 µM CBN. This is notable for two reasons. First, as the inactivation *V*_1/2_ of Nav1.7 is hyperpolarized relative to the RMP of DRG neurons (when there is no action potential (AP) firing), a substantial fraction of the membrane-bound Nav1.7 channels at RMP are in an inactivated-state, more closely fitting with the data shown in Fig. 4d than Fig. 4b, which suggests that under physiological conditions, relatively lower concentrations of CBN would prevent Nav1.7 channels from opening. Second, as more slow inactivation is reached, these results suggest that CBN can inhibit Nav channel activity at even lower concentrations than suggested by the steady-state voltage-dependence data shown in Fig. 3.

We show **τ**_Slow_ and fraction of the recovery fit with **τ**_Slow_ in Fig. 4e, f. These data indicate that CBN increases the slower fraction of recovery, while also increasing the time constant of the slow component of recovery from inactivation from all the tested pre-pulse durations. This suggests that CBN slows recovery from inactivation, which supports the hypothesis that CBN stabilizes the slower inactivated states of the channel; as noted above, CBN does this at lower concentrations (e.g., 1 vs. 15 µM) than would be expected based on steady-state measurements. These observations are similar to those we found previously with CBD and CBG^20,21,27^, with one major difference, i.e., that the noted compounds imparted a similar impact on both faster as well as slow inactivated channels. Although the slowing of recovery from inactivation for CBD and CBG also became more pronounced in slow inactivation, CBN seems to only select for slow inactivation, which is elicited over the time course of seconds.

### CBN hyperpolarizes steady-state slow inactivation

**Fig. 5:**
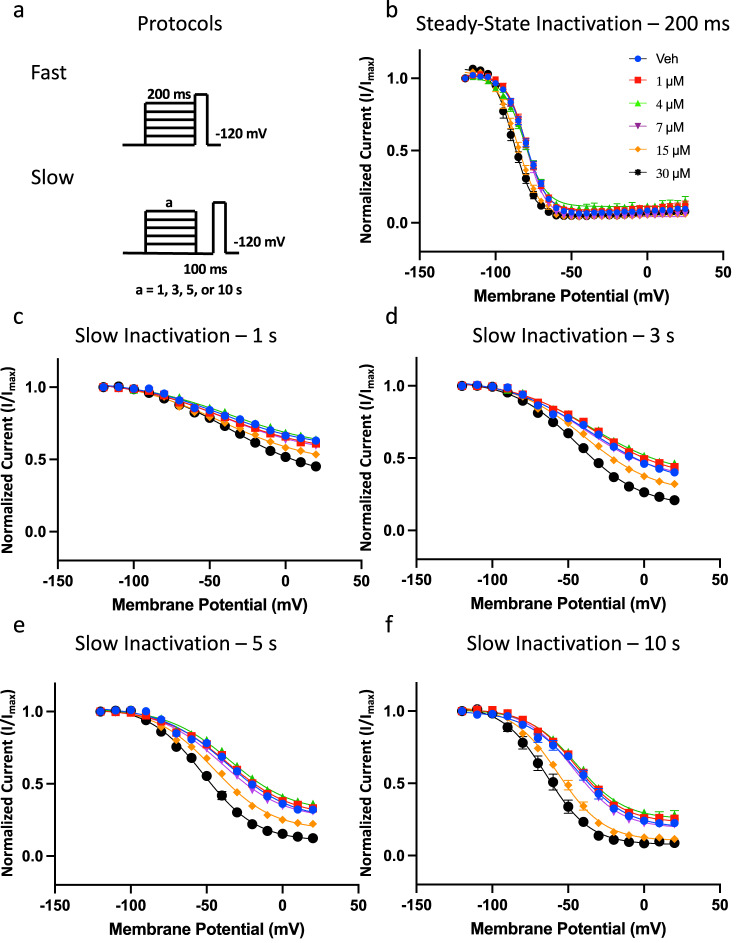
CBN hyperpolarizes slow inactivation curves in Nav1.7, imparts similar effect from a 200 ms pre-pulse. **a** Shows the protocols that were used to measure CBN’s effect on inactivating properties. The top protocol was used to measure SSI from 200 ms, and the bottom protocol was used to measure steady-state slow inactivation (SSSI) from varying durations. **b** 200 ms inactivating probability curves at Veh vs. different CBN concentrations. Midpoints (in mV). **c** SSSI from 1 s, **d** 3 s, **e** 5 s, and **f** 10 s. Data shown as means ± SEM (*n* = 11–17 for 200 ms, 18–24 for 1 s, 15–22 for 3 s, 11–20 for 5 s, and 7–13 for 10 s).

To determine whether CBN’s hyperpolarizing effect on inactivation also occurs during shorter pre-pulse durations, we performed another set of experiments at 200 ms^21,29,30^ (Fig. 5a, b). In line with the channels accumulating more inactivation with longer depolarization durations at each step, Nav1.7 presented a more depolarized inactivation curve at 200 ms (−79.9 ± 0.3 mV) than 500 ms (−84.6 ± 1.4 mV) (Figs. 5b,  3c, Table S1). These experiments indicate that, similar to 500 ms, at 200 ms, CBN imparts similar effects on the inactivation curve. The CBN effect on the SSI curve became significantly different from vehicle at 15 µM (*p* < 0.05) (Fig. 5b).

Then, we evaluated the impact of CBN on steady-state slow inactivation at 1, 3, 5, and 10 s durations (Fig. 5c–f). We first held channels at −120 mV, which was followed by depolarizing pulses for one of several noted time courses. This was followed by a hyperpolarizing pulse back to −120 mV for 100 ms to recover the channels that were fast inactivated. The choice of 100 ms for recovery was based on the earlier experiments shown in Fig. 4b. The current amplitude was measured using a test-pulse to −20 mV (Fig. 5). Consistent with our previous findings (i.e., recovery from 5 s of inactivation in Fig. 4d), we found that CBN concentration-dependently also stabilizes the steady-state slow inactivated states of Nav1.7, and that this effect becomes more pronounced as the channels enter deeper inactivated states (i.e., more so after 10 s than 3 s^24^), (Fig. 5, Table S1). These data further suggest that as channels accumulate more inactivation, CBN induces more pronounced effects on Nav1.7 gating, and that CBN seems to select for slow inactivation.

### CBN hyperpolarizes the SSI curve in Nav1.7-P610T

We recently characterized a mutation in Nav1.7, P610T, that was discovered in two siblings with persistent ocular pain post corneal axonal transection^30^. We found that P610T impairs slow inactivation in Nav1.7, thereby causing hyperexcitability in neurons. As CBN seems to select for slow inactivation, we tested its effects on P610T and WT channels, using a 5 s pre-pulse duration. As expected, CBN hyperpolarized the SSI curves in both WT and P610T (Fig. S1); however, the magnitude of the shift was larger in the mutant variant (*p* < 0.05).

### CBN equipotently inhibits apparent conductance and hyperpolarizes inactivation

**Fig. 6:**
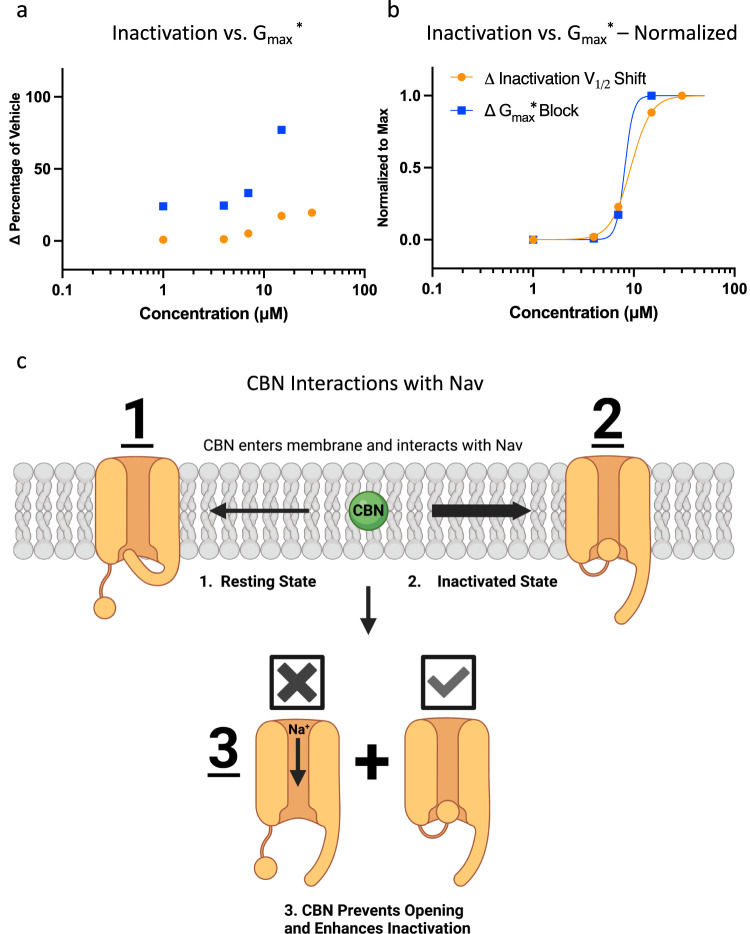
CBN effect on apparent conductance compared to its effect on inactivation. **a** Comparison of concentration-dependent effects of CBN on *G*_max_^*^ vs. inactivation as a percentage of vehicle. **b** Normalized relationship of the data from **a** and fit with the Hill equation. The Hill coefficient was not constrained during fitting. **c** Cartoon representation of the concentration-dependent modality of CBN’s modulation of voltage-dependent Nav currents. Given than CBN is hydrophobic, it readily partitions into the membrane. Once inside the membrane it interacts to a very small extent with the (1) resting state and a much larger affinity for the (2) inactivated states of the Nav channel. (3) CBN equipotently prevents channels from opening, as it hyperpolarizes/enhances inactivation hence.

We previously described pharmacological consequences of targeting *G*_max_^*^ vs. stabilizing inactivation^4,21^. In these earlier studies we determined that CBG tends to target *G*_max_^*^ more potently than channel inactivation. Here, we applied the same analysis to CBN. As shown in Figs. 2 and 3, CBN’s effects on both parameters became statistically significant at 15 µM. The quantification of these effects was done by first subtracting mean numbers across various concentrations of CBN from the mean numbers from vehicle that are shown in Fig. 2b (*G*_max_^*^) and Fig. 3d (Inactivation *V*_1/2_). This calculation yields the ∆ between CBN effects at a given concentration versus vehicle. The ∆ was then divided by the results in vehicle to obtain the ∆percentage of vehicle. The results are plotted in Fig. 6a, which show that CBN alters both the Nav1.7 *G*_max_^*^ and inactivation *V*_1/2_. Interestingly, by the time 15 µM CBN is reached, most of the *G*_max_^*^ is inhibited.

We normalized the numbers in Fig. 6a to the maximal values, and plotted those values in Fig. 6b to get an approximation of the potency difference between the two effects. This normalization is based on a rationale that if *G*_max_^*^ is mostly, or entirely blocked, then any further shift in inactivation would be of little to no consequence. For instance, ∆percentage shift of *V*_1/2_ at the maximal concentration of 30 µM is only at about 20% of vehicle, because 15–30 µM abolishes ~80% of *G*_max_^*^, any further shifts (which is unlikely to be large, Fig. 3) in *V*_1/2_ would be physiologically insignificant. Fitting the data with the Hill equation indicated that CBN’s IC_50_ in inhibiting *G*_max_^*^ is estimated to 8.2 µM, and the IC_50_ for hyperpolarizing *V*_1/2_ is approximated to 9.3 µM.

CBN, like other cannabinoids, is highly hydrophobic (a higher partitioning/distribution of compound in lipid vs. water indicates a greater preference for the membrane phase at equilibrium). This property enables the compound to readily penetrate the membrane and interact with the inactivated states of Nav channels. Because RMP in DRG neurons is considerably more depolarized than Nav1.7’s availability *V*_1/2_, lower concentrations of CBN in the membrane could be sufficient to keep channels in a state of inactivation, which would culminate in a reduction of neuronal excitability (Fig. 3c). CBN’s relative non-selectivity in targeting *G*_max_^*^ vs. inactivation suggests that its inhibition of Nav may be driven by stabilization of inactivation and not activation (consistently with Fig. 2) and could be primarily due to indirect non-selective modulation of the local membrane environment^27,31^ (Fig. 6c).

### CBN reduces spontaneous excitability in rat DRG neurons

**Fig. 7:**
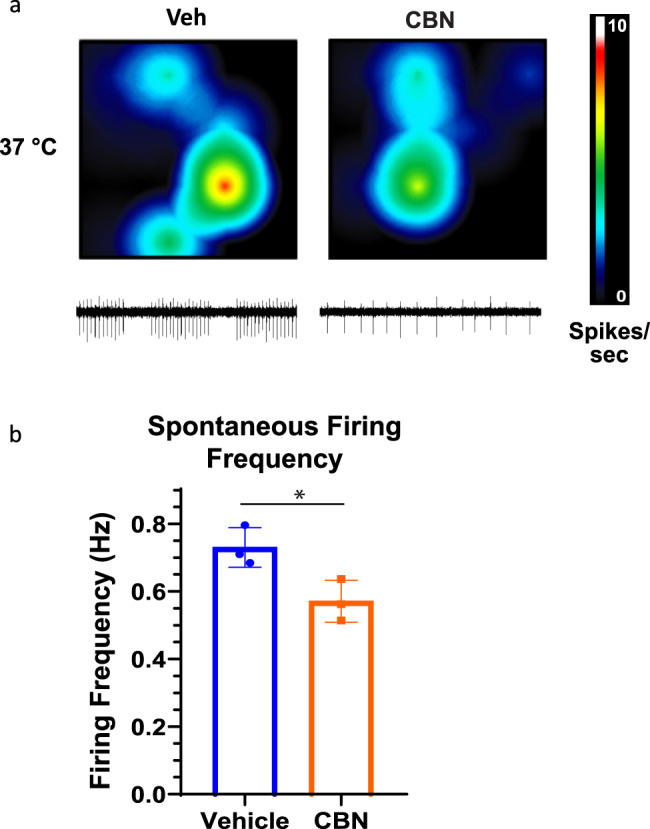
CBN reduces spontaneous excitable activity of rat DRG neurons in MEA. **a** Representative images of MEA recordings of AP firing at vehicle and 10 µM CBN (picked from concentration-response relationships in Fig. 1). The firing frequency of each active electrode is color coded: white/red mean high, and blue/black mean low frequencies. **b** Quantification of MEA data showing firing rate (*n* = 3 for each). * Indicates *p* < 0.05.

To determine whether CBN inhibits neuronal activity, we measured excitability of rat DRG neurons using multielectrode array recordings (MEA). We measured spontaneous firing over a 10-minute period in MEA wells that had vehicle and 10 µM CBN (Fig. 7a, b) (the activity of all wells was compared before vehicle/CBN addition; there was no significant difference between the wells). Our results suggested that CBN reduces DRG neuronal firing. These findings suggest that CBN reduces neuronal firing at concentrations similar to those where it inhibits Nav currents (Fig. 1b). Therefore, given that Nav channels are vital to excitability, CBN’s inhibition of firing could be driven, at least in part, via inhibition of Navs.

### CBN inhibits native nociceptive sodium currents in freshly isolated DRG neurons

**Fig. 8:**
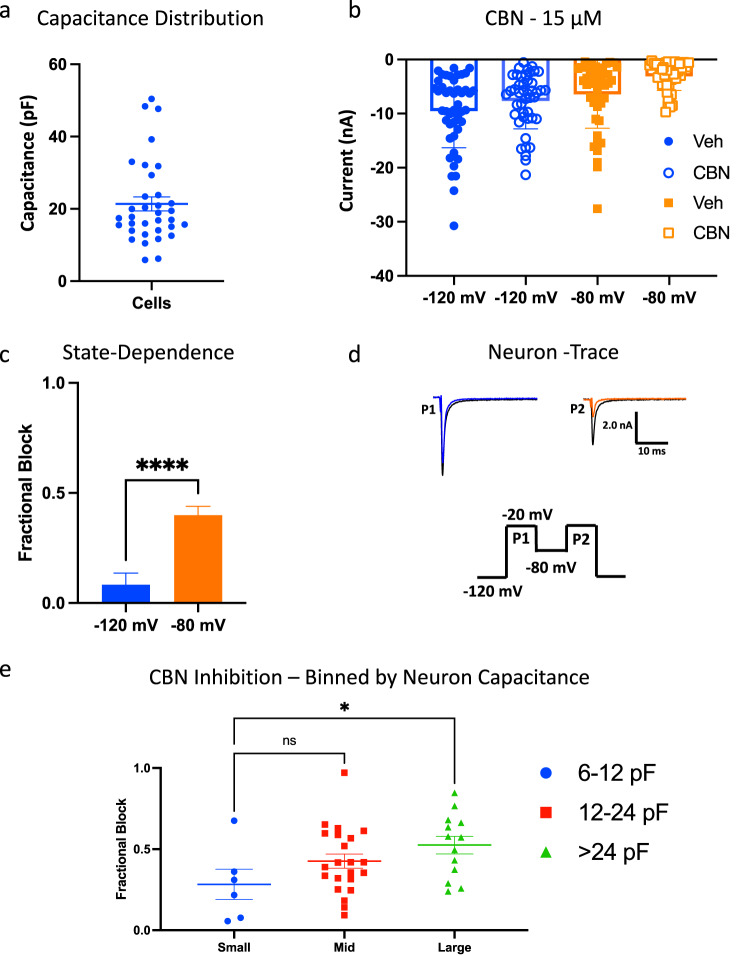
CBN inhibits native voltage-dependent sodium currents in freshly isolated DRG neurons. **a** The distribution of the capacitance sizes we got from our cohort of neurons. **b** Mean peak amplitudes of neuronal Nav currents from a holding-potential of −120 and −80 mV, in vehicle and 15 µM CBN. All measurements are matched-paired. **c** CBN’s potency across all neurons at −120 and −80 mV shown as fractional block. Data shown as means ± SEM (*n* = 41). **d** The two-pulse protocol that was used to measure inhibition, along with a representative trace from each of P1 and P2. **e** The cohort of neurons binned by capacitance sizes. Data shown as means ± SEM (*n* = 41). * Indicates *p* < 0.05, **** indicates *p* < 0.0001, ns indicates not significantly different *p* > 0.05.

DRG neurons are known to express complex and diverse ensembles of ion channels and receptors. Specifically, DRG neurons across various diameters express different stoichiometric combinations of Nav channels, with characteristic biophysical signatures (e.g., kinetic and voltage-dependent properties). We recently developed a new robotic automated high-throughput method to investigate the biophysical properties of diverse neurons, compared head-to-head and simultaneously, immediately after dissociation and isolation of cells from intact animal tissue^32^. We used this method to investigate the effects of 15 µM CBN on primary DRG neurons across various sizes obtained from rat pups. We used cell capacitance as a proxy for neuronal diameter. The cohort of cells in our experiments ranged from ~6 to 50 pF in size (Fig. 8a). To measure CBN effects, we designed a protocol to simultaneously measure inhibition from full rest and after accumulation of some inactivation in most Nav channels. We first held channels at −120 mV for 50 ms, then pulsed to (P1) −20 mV for 20 ms, followed by a 100 ms conditioning pulse at −80 mV, which preceded another 20 ms pulse to (P2) −20 mV, followed by a final recovery pulse back to −120 mV for another 50 ms, before the next cycle (Fig. 8a–d). Like our previous experiments, we found that CBN inhibits the native sodium currents more potently when the membrane potential is clamped at a more depolarized potential (Fig. 8a–c). The individual constituent Nav channels in each neuron include various proportions of Nav1.1/6-9. Therefore, these results suggest that CBN is a structurally non-selective inhibitor of nociceptive Nav channels.

As small and large diameter neurons are known to express different combinations of Nav channels^33–36^, we sought to determine if CBN imparts inhibition in a differential manner across different neuron diameters. Studies suggest that Nav1.7 and Nav1.8 are expressed within DRG neurons across a range of different diameters; however, the smaller neurons generally express a substantial proportion of tetrodotoxin-resistant (TTX-R) (e.g., Nav1.8/9) with the larger neurons expressing tetrodotoxin-sensitive (TTX-S) (e.g., Nav1.6) channels. Among these channels, Nav1.8 has the most depolarized availability *V*_1/2_, and Nav1.7 has the most hyperpolarized, with Nav1.6 and Nav1.9 falling in between. Based on the distribution of capacitances in our cohort of neurons, we divided the cells into three capacitive bins (2-fold breakdown): 6–12 pF (small), 12–24 pF (mid), and >24 pF (large) (Fig. 8e). Because CBN does not produce much inhibition from resting state (Fig. 1), this analysis was done only at the −80 mV pulse, where Nav1.8 channels are mostly at rest (P2) but the other Navs are not. Expectedly, there was variability among the neuronal response to CBN; however, the largest neurons on average were inhibited more than the smallest neurons (*p* < 0.05). This is an intriguing finding, as Nav1.8, with an inactivation *V*_1/2_ of ~−30 mV, is considered to be more prominent among the smaller neurons. From a holding-potential of −80 mV, the majority of Nav1.8 would be at rest, and hence less responsive to CBN which selects for inactivation. These results demonstrate the functional selectivity of CBN on diverse Nav channels in a simultaneous and head-to-head manner in a cohort of primary neurons, in real time.

To directly assess CBN’s potential structural selectivity on Nav channels, we performed a series of experiments in which CBN was added to DRG neurons that were perfused with 500 nM TTX (Fig. S2). In these experiments we patch-clamped cells with capacitances that ranged from ~5–25 pF (Fig. S2a). As expected from the IV relationships, we found that in the presence of both TTX and CBN, from −90 mV (when the TTX-R Nav1.8 is expected to be mostly at rest), CBN did not inhibit the Nav currents (*p* > 0.05), however, from −55 mV, when Nav1.8 accumulates more inactivation, CBN significantly inhibited remaining macroscopic Nav currents (Fig. S2b) (*p* = 0.0037). This suggests that a large component of the TTX-R currents in our cohort of cells were Nav1.8, and thus implies that CBN is likely not a structurally selective Nav channel inhibitor.

**Fig. 9:**
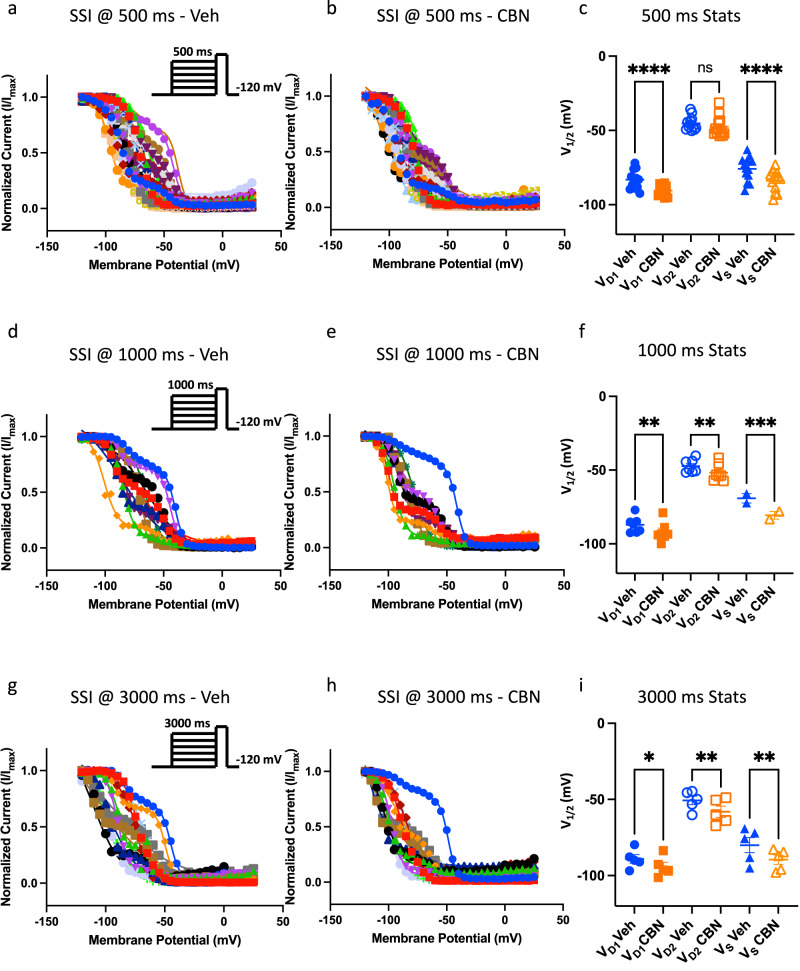
CBN hyperpolarizes inactivation curves in freshly isolated DRG neurons. **a**–**c** All measurements are matched-paired. SSI measured from a 500 ms pre-pulse duration. Double Boltzmann curve midpoints (*V*_D1_ and *V*_D2_) are shown in **c**. **d**–**f** Data from 1000 ms, and **g**–**i** from 3000 ms durations. **c**, **f**, **i** Data shown as means ± SEM (*n* = 34 for 500 ms, 12 for 1000 ms, and 16 for 3000 ms) *V*_D1_ refers to the first *V*_1/2_ and *V*_D2_ to second *V*_1/2_ of double Boltzmann fits, and *V*_S_ refers to the singular *V*_1/2_ of single Boltzmann fits.

Next, to further describe CBN’s stabilization of inactivation, we measured its effects on steady-state inactivation from 500, 1000, and 3000 ms pre-pulse durations in the same cohort of neurons. Expectedly, most of the neurons displayed inactivating probability curves that are well-described with a double Boltzmann fit, which is indicative of the macroscopic Nav current being composed of different Nav channels. However, some cells were better fit with a single Boltzmann. At 500 ms, CBN significantly hyperpolarized the more negative midpoint (V_D1_) from the cells that were better fit a double Boltzmann (*p* < 0.0001), but not the second more positive midpoint (V_D2_) (p > 0.05). CBN also significantly hyperpolarized the cells that were better fit a single Boltzmann (*p* < 0.0001) (Fig. 9a–c). CBN significantly hyperpolarized all three midpoints after longer pre-pulse durations (deeper inactivation) of 1000 and 3000 ms (Fig. 9d–i). As noted, each of these three midpoints are indicative of different amounts and combinations of Nav channels; therefore, the lack of statistical significance at the most depolarized midpoint (V_D2_), which is likely dominated by Nav1.8^32^, after shortest duration of 500 ms, suggests that CBN functionally targets and stabilizes Nav inactivation, which inhibits Nav currents. This further suggests that it does not have structural selectivity among Nav channels.

**Fig. 10:**
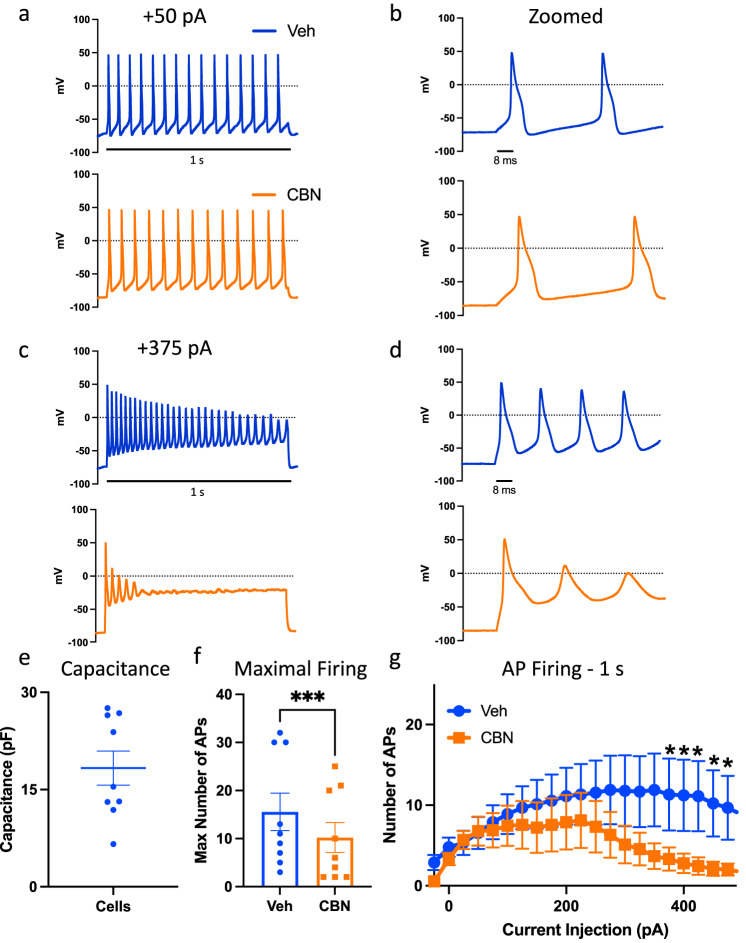
CBN inhibits triggered excitability. **a**–**d** All measurements are matched-paired. Sample action potential traces at +50 and +375 pA current injections. **e** Cell capacitance distribution. **f** Maximal number of action potentials in Veh vs. CBN. **g** Collected results for the number of action potentials during 1 s of current injections in Veh vs. CBN (15 µM). Data shown as means ± SEM (*n* = 9). * Indicates *p* < 0.05.

Finally, we sought to determine if CBN also inhibits triggered excitability the same way it inhibited spontaneous activity in MEA recordings. To do this, we used a standard step-wise current injection protocol in current-clamp. This analysis indicates that CBN indeed inhibits the firing of freshly isolated DRG neurons (Fig. 10).

## Discussion

The cannabis plant and its biologically active constituents have a long history of being used as therapeutics. Phytocannabinoids and terpenes have been investigated for a wide range of hyperexcitability disorders, and a large body of evidence shows that CBD and CBG modulate the function of many receptor proteins, as well as the biophysical properties of the membrane in which those proteins are embedded^1,4,12,37^. Given the similarities in the physicochemical properties of many cannabinoids, the noted findings suggest that the CB-independent pathways could be vital to their physiological pharmacology. We recently discovered that these pharmacological properties (combination of CB-dependent and -independent pathways via Nav channels), for instance in CBG, may contribute to its analgesic properties^21,38^. As animal studies have suggested that CBN also possesses analgesic properties^6^, in this study we first demonstrate its in-depth effects on Nav1.7, which is a major driver of firing in nociceptive neurons, and then took our results in Nav1.7 as a guiding principle to investigate CBN effects on native sodium currents and action potential firing in native DRG neurons. We found that the apparent potency of CBN at the most depolarized holding-potential (among the three we tested in Fig. 1) of −90 mV was 10.7 µM, whereas the CBN effects on *G*_max_^*^ and SSI became significant at 15 µM (Figs. 2b, 3d). We conducted the experiments on neuronal spontaneous activity (MEA) at 10 µM, when all of the intact cells are at normal RMP, which is a depolarized potential compared to the availability curve of Nav1.7, while the gating experiments on DRG neurons were performed at 15 µM. As previous rodent studies suggest that CBD’s efficacious plasma levels are within ~8.5 µM range^39^, given the similarities between CBD and CBN, our results indicating CBN’s inhibition of Nav currents and excitability at the concentrations of 7–15 µM are concordant.

The use of cannabinoids for treating various types of pain is not a novel idea. For instance, potent CB receptor agonists were suggested to reduce the severity of chemotherapy-induced neuropathy^40,41^. However, the role of cannabinoids in treatment of pain is likely to be complex, and it has been suggested that excessive activity at these receptors might exacerbate symptoms^42–46^. Conversely, in vivo treatment of CBD has robustly reduced chemo-induced neuropathic symptoms. The clinical results associated with testing Sativex (a 1:1 mixture of CBD and THC) against neuropathic pain^46–48^, previously prompted us to suggest that pain relief might be achieved via the use of compounds that have some affinity for the CB receptors, but also work through CB-independent pathways. Thus, we argued that CBG may possess the favorable features of both pathways, without the unwanted effects of strong agonism at the CB receptors (e.g., THC). Like CBG, CBN also seems to work through both pathways. However, CBN has a stronger affinity for the CB-dependent pathway than CBG, but is still non-psychoactive. Therefore, if one places CBD on one end, and THC on the other end of the spectrum of CB-independent to CB-dependent pathways, CBG would fall closer to the CBD end, and CBN would be closer to the THC end. Therefore, the question remains whether being closer to either end of the noted spectrum would trigger a greater response for pain therapy. Indeed, rat studies suggest that a 1:1 ratio of CBD to CBN (at 1 mg/ml each) could enhance analgesic relief^6^.

Finally, our goal in this study was to investigate CBN with respect to Nav channels. The in-depth biophysical and pharmacological descriptions in this paper provides an in vitro characterization of the effects of CBN on both heterologously and natively expressed Nav channels in DRG neurons. Thus, our results contribute to understanding of CBN’s effects on peripheral mechanisms of pain signaling. A full understanding of CBN’s effects on pain will require dissection of the long biological pathway that reaches from the peripheral nervous system (PNS) to the central nervous system (CNS). CBN’s mechanism of action on each individual component and step of the pain pathway (as well as other physiological experiences) will require further study. Safety aspects of the potential application of CBN as a therapeutic, and necessary mode of administration (including systemic application, etc.) would need to be investigated separately, as CBN likely modulates physiological functions via multiple mechanisms, some of which have not been described in this study on DRG neurons.

From a Nav channel perspective, several points deserve consideration. First, Nav channels (notably Nav1.7) are critical to pain signaling^15,17,49,50^; however, effective target engagement using selective small molecules has thus far been elusive^51^. To circumvent these issues, higher doses would be needed, which cause off-target effects^52^ (cardiac toxicity is particularly problematic). Cannabinoids have the advantage of being relatively well-tolerated^37^, which is likely due to their highly hydrophobic nature (calculated-LogD for CBD, CBG, and CBN are 6.60, 7.04, and 6.41). Their hydrophobic nature would increase likelihood of accumulation in the membranes of tissues that have higher lipid contents (e.g., nerves vs. heart)^21,53,54^. This would suggest that cannabinoids could achieve sufficient engagement with Nav channels in DRG neurons (assuming an appropriate mode of administration to minimize distribution in other tissues). Given that all three phytocannabinoids (CBD, CBG, and CBN) are state-dependent inhibitors of Nav currents^4,20,21,55^, a given concentration of these compounds would be expected to affect Nav1.7 more potently than other Nav channels within DRG neurons^4^. This inhibition of Nav currents could work in concert, to varying degrees (depending on the cannabinoid), with interactions at CB receptors to effectively reduce pain. As Nav1.7 is thought to be expressed across various classes of varying-diameters of DRG neurons^56^, CBN’s preference for slow inactivation and Nav1.7 would be expected to shape its effect on a spectrum of cells that express Nav1.7. The stoichiometry of Nav channel expression as a function of total macroscopic Nav current within a given cell (including Nav1.7) would play a major role in determining the effect of CBN.

Outside of the sodium channel and endocannabinoid receptor paradigms, CBN has been shown to be a robust agonist of TRPA1 and an antagonist of TRPM8 channels^2,13^. CBN’s potential therapeutic application to pain will undoubtedly be shaped by its effect on these channels which have vital roles in pain and thermo-sensation^57^. Future functional studies of this compound will need to examine the different stoichiometric and ensembles of all of these channels and receptors in different types of neurons within pain pathways.

Based on the theory in which both CB-dependent and -independent pathways should be present for ideally treating pain, as CBG and CBN fall in the middle of the spectrum, they would be expected to be superior to either of CBD or THC. We previously found that CBG inhibited *G*_max_^*^ more potently than it stabilized inactivation^21^. We argued that this property could prevent a nociceptive firing event from starting in the first place, and that this would be favorable for pain therapy, as it would suggest prevention of pain from initiating rather than reducing its severity. Here, we found that CBN equipotently affects both *G*_max_^*^ and inactivation; from this perspective CBN would be an inferior compound relative to CBG. Our results also suggest that CBN has a stronger preference for slow inactivated Nav1.7 channels compared to CBG. CBN barely inhibited Nav1.7 from a holding-potential of −110 mV (when most channels are rest), and hyperpolarized the inactivation curves as the duration of pulses became longer at elevated concentrations. From this perspective, CBN may be superior to CBG, as Nav1.7 is mostly inactivated at RMP in DRG neurons, and having a compound that almost entirely selects for the most inactivated states of the channels could suggest a greater functional selectivity for Nav1.7. Indeed, our simultaneous head-to-head recordings from diverse freshly isolated neurons provide a picture of CBN’s functional selectivity in action, and suggest different degrees of inhibition in different groups of cells. We have also recently identified Nav1.7 mutations associated with some painful disorders, including ophthalmic pain^30,58^, that only disrupt slow inactivation. This suggests that CBN or CBN-like compounds may be well-suited for restoring the function of such mutations, especially considering CBN is already being developed for eye-related problems^8^. Indeed, our experimental data describing the effects of CBN on the Nav1.7-P610T, a mutant variant in Nav1.7 associated with ophthalmic pain, supports this idea.

Our results suggest that CBN is slightly less potent in inhibition of Nav1.7 than either CBD or CBG. Furthermore, given that CBN equipotently affects *G*_max_^*^ and inactivation, we suggest that CBN’s inhibitory effects on sodium currents could be predominantly allosteric/indirect in nature. In a series of functional^20,27^, structural^59^, and molecular dynamics-based^27^ studies, we previously discovered that CBD’s inhibition of Nav currents has at least two components: (1) *G*_max_^*^ block arising from direct occlusion of the Nav pore at the fenestration interface, and (2) stabilization of inactivation arising, in part, through altering membrane stiffness. The membrane effect has been extensively investigated using amphiphilic compounds, which typically at elevated concentrations display promiscuity of receptor targets via changing membrane properties, and were shown to not affect Nav activation and hyperpolarize inactivation^1,4,31,60,61^. These features are similar to CBN’s effects on Nav1.7. CBN’s equipotency in affecting *G*_max_^*^ and inactivation also suggests that its inhibition is likely primarily driven by stabilizing inactivation, and not directly blocking the pore, affecting *G*_max_ or activation.

Pharmacological assessment of agents that act on channels or receptors has traditionally been based on patch-clamp study, which permits analysis in voltage-clamp or current-clamp mode^62,63^, but is limited by the need to record using electrodes that involve perfusion of intracellular contents and, when carried out in manual mode, by inherent constraints on throughput. MEA recordings in intact neurons measure activity recorded extracellularly from larger numbers of neurons, but do not provide an assessment of action potentials. Constraints on the throughput of manual patch-clamp have been overcome using automated patch-clamping^20,21,32,52^. However, as usually performed, automated patch-clamp analysis is generally used to assess ion channels or receptors expressed in cell lines, and is not practicable for native neurons; this is particularly important since the properties of ion channels can be different following expression in cell lines versus expression within neurons^64^. In the present study, we assessed the effects of CBN on Nav1.7 and on DRG neuron excitability using these three approaches. In addition to the automated patch-clamp analysis of Nav1.7 channels expressed in a cell line and MEA analysis of DRG neurons, we used a newly developed method for automated high-throughput patch-clamp analysis that permits study of freshly isolated DRG neurons^32^. In addition to providing a basis for high-throughput analysis of multiple cells in a simultaneous comparative manner, this new methodology permits assessment of these cells by both voltage-clamp (Figs. 8, 9) and current-clamp (Fig. 10). As illustrated by this study, this new methodology can be used together with traditional automated patch-clamp methods that record from cell lines, or MEA, for a more comprehensive assessment of the effects of pharmacological agents on ion channels expressed within the neuronal cell types that normally express them. This is especially important given the strong effect of cell background on the properties of ion channels^64^.

In conclusion, the results of this study support the idea that CBN acts to inhibit Nav channels in a functionally selective manner, an action that could work in concert with its interactions at CB receptors. CBN’s impact on Nav currents is via influencing both the voltage-dependence of slow inactivation and recovery from slow inactivation, but without impacting *G*_max_ or fast inactivation. The combined result of these effects along with interactions at other targets could contribute to its analgesic effects. CBN’s inhibitory effects on Nav currents and DRG neuron excitability give its actions a new dimension and raise the possibility that this cannabinoid may be effective for treating neuropathic pain.

## Methods

### Cell culture

Human Embryonic Kidney (HEK293) (CLS Cat# 300192/ p777_HEK293, RRID:CVCL_0045) cells were used for automated patch-clamp experiments. HEK293 cells were stably transfected with human Nav1.7 channels. The human *SCN1B* cDNA construct was transfected into the cell line. All cells were incubated at 37 °C/5% CO_2_. Lipofectamine 2000 was used for transfection of HEK293 cells with WT or P610T plasmids with total of 10 µg cDNA.

Primary sensory neuron isolation for electrophysiological animal studies followed a protocol approved by the Department of Veterans Affairs West Haven Hospital Animal Use Committee.

The patch-clamp recordings from freshly isolated DRG neurons were performed in cells from rat pups. DRGs were harvested, dissociated, isolated, and used in suspension form for the experiments described below. The protocol was described in Ghovanloo et al.^32,65^.

### Automated patch-clamp

Automated patch-clamp recording was used for study of the effects of CBN on Nav1.7 in HEK293 cells as previously described^21^. Sodium currents were measured in the whole-cell configuration using a Qube-384 (Sophion A/S, Copenhagen, Denmark) automated voltage-clamp system. Intracellular solution contained (in mM): 120 CsF (or KF for CC experiments), 10 NaCl, 2 MgCl_2_, 10 HEPES, adjusted to pH7.2 with CsOH. The extracellular recording solution contained (in mM): 145 NaCl, 3 KCl, 1 MgCl_2_, 1.5 CaCl_2_, 10 HEPES, adjusted to pH7.4 with NaOH. Liquid junction potentials calculated to be ~7 mV were not adjusted for. Currents were low pass filtered at 5 kHz and recorded at 25 kHz sampling frequency. Series resistance compensation was applied at 100% and leak subtraction enabled. The Qube-384 temperature controller was used to maintain recording chamber temperature for all experiments at 22 ± 2 °C at the recording chamber. Appropriate filters for cell membrane resistances, series resistance (<10 MOhm) and Nav current magnitude (>500 pA (in HEK cells) at a test pulse from a resting HP of −120 mV) were routinely applied to exclude poor quality recordings. Vehicle controls were run on each plate to enable correction for any compound-independent decrease of currents over time. Baselines were established after 20 minutes in vehicle. Fractional inhibition was measured as current amplitude from baseline to maximal inhibition after 20-minute exposure to test compound unless otherwise noted. Normalized mean inhibition data were fit to the Hill-Langmuir equation:

1 $${{{\rm{Y}}}}={[{{{\rm{C}}}}]}^{{{{\rm{h}}}}}/({{{{{\rm{IC}}}}}_{50}}^{{{{\rm{h}}}}}+{[{{{\rm{C}}}}]}^{{{{\rm{h}}}}})$$

To estimate the half maximal inhibitory concentration (IC_50_ value); where Y is the normalized inhibition, C the compound concentration, IC_50_ the concentration of test compound to inhibit the currents 50%, and h the Hill coefficient. Data analysis was performed using Analyzer (Sophion A/S, Copenhagen, Denmark) and Prism (GraphPad Software Inc., La Jolla, CA, USA) software. All HEK voltage-clamp experiments were done using the Qube.

Automated patch-clamp was used to assess freshly isolated, native DRG neurons, using methods developed by Ghovanloo et al.^32^. Appropriate filters (as described in Ghovanloo et al.^32^) and solutions, including high Ca^2+^ saline solution to enhance seal formation were used.

### Compound preparation

CBN was purchased from Cayman Chemicals. Powdered CBN was dissolved in 100% DMSO to create stock. The stock was used to prepare drug solutions in extracellular solutions at various concentrations with no more than 0.5% total DMSO content.

### Activation and conductance protocols

To determine the voltage-dependence of activation, we measured the peak current amplitude at test pulse potentials ranging from −120 mV to +25 mV in increments of +5 mV for 500 ms. Channel conductance (G) was calculated from peak I_Na_:

2 $${{{{\rm{G}}}}}_{{{{\rm{Na}}}}}={{{{\rm{I}}}}}_{{{{\rm{Na}}}}}/({{{\rm{V}}}}-{{{{\rm{E}}}}}_{{{{\rm{Na}}}}})$$

where G_Na_ is conductance, I_Na_ is peak sodium current in response to the command potential V, and E_Na_ (measured on IV relationships) is the Nernst equilibrium potential. Calculated values for conductance were fit with the Boltzmann equation:

3 $${{{\rm{G}}}}/{{{{\rm{G}}}}}_{\max }=1/(1+\exp [{{{{\rm{V}}}}}_{1/2}-{{{{\rm{V}}}}}_{{{{\rm{m}}}}}]/{{{\rm{k}}}})$$

where *G*/*G*_max_ is normalized conductance amplitude, *V*_m_ is the command potential, *V*_1/2_ is the midpoint voltage and *k* is the slope.

To measure drug effect on conductance, we made measurements obtained across a range of membrane potentials using the noted step-pulse protocol. The membrane potential that elicited the maximal conductance (*G*_max_) was determined from this range. The drug effect was determined by measuring *G*_max_ in the presence and absence of the drug.

### Steady-state inactivation protocols

The voltage-dependence of fast/intermediate inactivation was measured by preconditioning the channels from −120 to +25 mV in increments of 5 mV for 3000/1000/500/200 ms, followed by a 20 ms test pulse during which the voltage was stepped to −20 mV (these protocols were used for HEK293 and/or DRG experiments). Normalized current amplitudes from the test pulse were fit as a function of voltage using the Boltzmann equation:

4 $${{{\rm{I}}}}/{{{{\rm{I}}}}}_{\max }=1/(1+\exp [{{{{\rm{V}}}}}_{1/2}-{{{{\rm{V}}}}}_{{{{\rm{m}}}}}]/{{{\rm{k}}}})$$

where I_max_ is the maximum test pulse current amplitude, at the most negative potential. The steady-state slow inactivation protocols involved step pulses from −120 mV to 20 mV for 1, 3, 5, or 10 s, followed by 100 ms recovery interval at −120 mV, followed by a test pulse to −20 mV.

### State-dependence protocols

To determine state-dependence, potency was measured from three different holding-potentials (−110, −100, −90 mV). The protocol started with a holding-potential of −110 mV followed by 180 × 20 ms depolarizing pulses to 0 mV at 1 Hz. Then, the holding-potential was depolarized by 10 mV, and the 180-pulse protocol was repeated at holding-potentials of −100 mV and −90 mV.

### Recovery from inactivation protocols

Recovery from inactivation was measured by holding the channels at −120 mV, followed by a depolarizing pulse to 0 mV, then the potential was returned to −120 mV. This was followed by a depolarizing 10 ms test pulse to 0 mV to measure availability. Recovery from inactivation was measured after pre-pulse durations of 20 ms, 500 ms, and 5 s and fit with a biexponential function of the form:

5 $${{{\rm{SpanFast}}}}=({\it{Y}}0-{{{\rm{Plateau}}}})\,\ast \,{{{\rm{PercentFast}}}}\,\ast \,0.01$$

6 $${{{\rm{SpanSlow}}}}=({\it{Y}}0-{{{\rm{Plateau}}}})\,\ast \,(100-{{{\rm{PercentFast}}}})\,\ast \,0.01$$

7 $${\it{Y}} = 	\,{{{\rm{Plateau}}}}+{{{\rm{SpanFast}}}}\,\ast \,\exp (-{{{\rm{KFast}}}}\,\ast \,{\it{t}})\\ 	 + {{{\rm{SpanSlow}}}}\,\ast \,\exp (-{{{\rm{KSlow}}}}\,\ast \,{\it{t}})$$

Where *t* is time in seconds, *Y*0 is the *Y* intercept at *t* = 0, KFast and KSlow are rate constants in units the reciprocal of *t*, PercentFast the fraction of the Y signal attributed to the fast-decaying component of the fit. Normalization was done by dividing peak current amplitude (test-pulse) by the peak (pre-pulse) current amplitude providing fraction amount of recovery as a function of time.

### Kinetics of inhibition

The kinetics of CBN block were measured at two potentials (when sizeable inhibition was observed) at 15 µM. The channels were held at respective holding-potentials followed by pulses to −20 mV. The inhibited sodium current was normalized and subsequently fit with a single exponential function:

8 $${{{\rm{Y}}}}=({{{\rm{Y}}}}0-{{{\rm{Plateau}}}})\,\ast \,\exp (-{{{\rm{K}}}}\,\ast \,{{{\rm{t}}}})+{{{\rm{Plateau}}}}$$

### Multielectrode array recordings

Multielectrode array (MEA) experiments were performed at 37 °C with a multi-well MEA system (Maestro, Axion Biosystems) according to a recently developed protocol^66^. Briefly, DRGs were isolated and cultured on MEA plates, maintained at 37 °C in a 5% CO_2_ incubator. A 24-well recording plate was used, embedded with a total of 16 electrodes per well. For each experiment, multiple wells were used to assess rat DRGs. Each well of the 24-well MEA plate (Axion Biosystems) was coated with poly-D-lysine (50 µg/ml) and laminin (10 µg/ml). MEA plates were read 72 hours after plating in the Axion Biosystems Maestro Multi-Well MEA system (Axion BioSystems, Atlanta, GA). The environment was allowed to equilibrate to 37 °C and 5% CO2 for 5 minutes prior to recording. Spontaneous DRG neuron firing activity was recorded for 10 minutes. Only neuron depolarizations (spikes) from active electrodes (defined as >1 spike/minute) were counted. The total number of spikes over the four wells from each condition was normalized by the number of active electrodes. The average of three biological replicates was taken.

### Statistics and reproducibility

Normalization was performed in order to control the variations in sodium channel expression and inward current amplitude and in order to be able to fit the recorded data with Boltzmann function (for voltage-dependences) or an exponential/biexponential function (for time courses of inactivation). The Sophion Qube is an automated electrophysiology instrument that is blinded to cell selections and experimentation, and selection is performed in an automated manner. All subsequent data filtering and analysis is performed in a non-biased manner, in which automated filters are applied to the entire dataset from a given Qube run. Fitting and graphing were done using Prism 9 software (Graphpad Software Inc., San Diego, CA) (PRISM, RRID:SCR_005375) (GraphPad, RRID:SCR_000306), unless otherwise noted. All statistical p-values report the results obtained from tests that compared experimental conditions to the control conditions. One-way analysis of variance (ANOVA): when multiple concentrations were each being compared to vehicle; or t-test: when overall 2 conditions were being compared. A level of significance *α* = 0.05 was used with p-values less than 0.05 being considered to be statistically significant. All values are reported as means ± standard error of means (SEM) or errors in fit, when appropriate, for *n* recordings/samples. Values are presented as mean ± SEM with probability levels less than 0.05 considered significant. The declared group size is the number of independent values, and that statistical analysis was done using these independent values.

### Reporting summary

Further information on research design is available in the Nature Portfolio Reporting Summary linked to this article.

## Supplementary information

SUPPLEMENTAL MATERIAL

Reporting Summary

## Data Availability

The datasets generated during and/or analyzed during this study are available from the corresponding author upon reasonable request. The numerical source files have been deposited in Dryad (10.5061/dryad.h70rxwdr2)^67^.
