# Supplementary material for: Functionally-selective inhibition of threshold sodium currents and excitability in dorsal root ganglion neurons by cannabinol
Source: Commun Biol. 2024 Jan 23;7:120. doi: 10.1038/s42003-024-05781-x (PMC10805714; doi:10.1038/s42003-024-05781-x)
Supplement: Supplementary file 1 — SUPPLEMENTAL MATERIAL [file 42003_2024_5781_MOESM1_ESM.pdf]

Table S1. $V_{1/2}$ (mV) for Activation, SSI 500 ms, SSI 200 ms, SI 1 s, SI 3 s, SI 5 s, and SI 10 s. These numbers are related to Figures 2, 3, and 4 ± SEM. * indicates statistical significance.

Concentration	Activation	SSI 500 ms	SSI 200 ms	SI 1 s	SI 3 s	SI 5 s	SI 10 s
**Veh**	-45.0±1.6	-84.6±1.4	-79.9±0.3	-41.2±6.4	-34.8±2.0	-36.9±1.6	-44.7±1.6
**1 µM**	-41.9±1.3	-84.0±1.5	-80.1±0.4	-42.1±3.8	-33.6±2.0	-36.1±1.1	-45.0±0.9
**4 µM**	-44.7±1.8	-86.1±1.9	-81.0±0.5	-36.2±4.5	-34.4±2.6	-34.8±1.8	-44.5±1.3
**7 µM**	-44.0±1.3	-89.0±3.6	-80.0±0.2	-40.9±4.4	-38.9±2.2	-40.1±1.4	-46.5±1.0
**15 µM**	-39.6±1.1	-99.3±1.3*	-85.6±0.4*	-42.0±3.8	-39.2±2.0*	-45.4±1.1*	-56.7±1.0*
**30 µM**	-48.4±6.5	-100.6±2.7*	-88.1±0.3*	-35.8±3.2	-44.2±1.4*	-52.4±1.0*	-64.4±1.4*

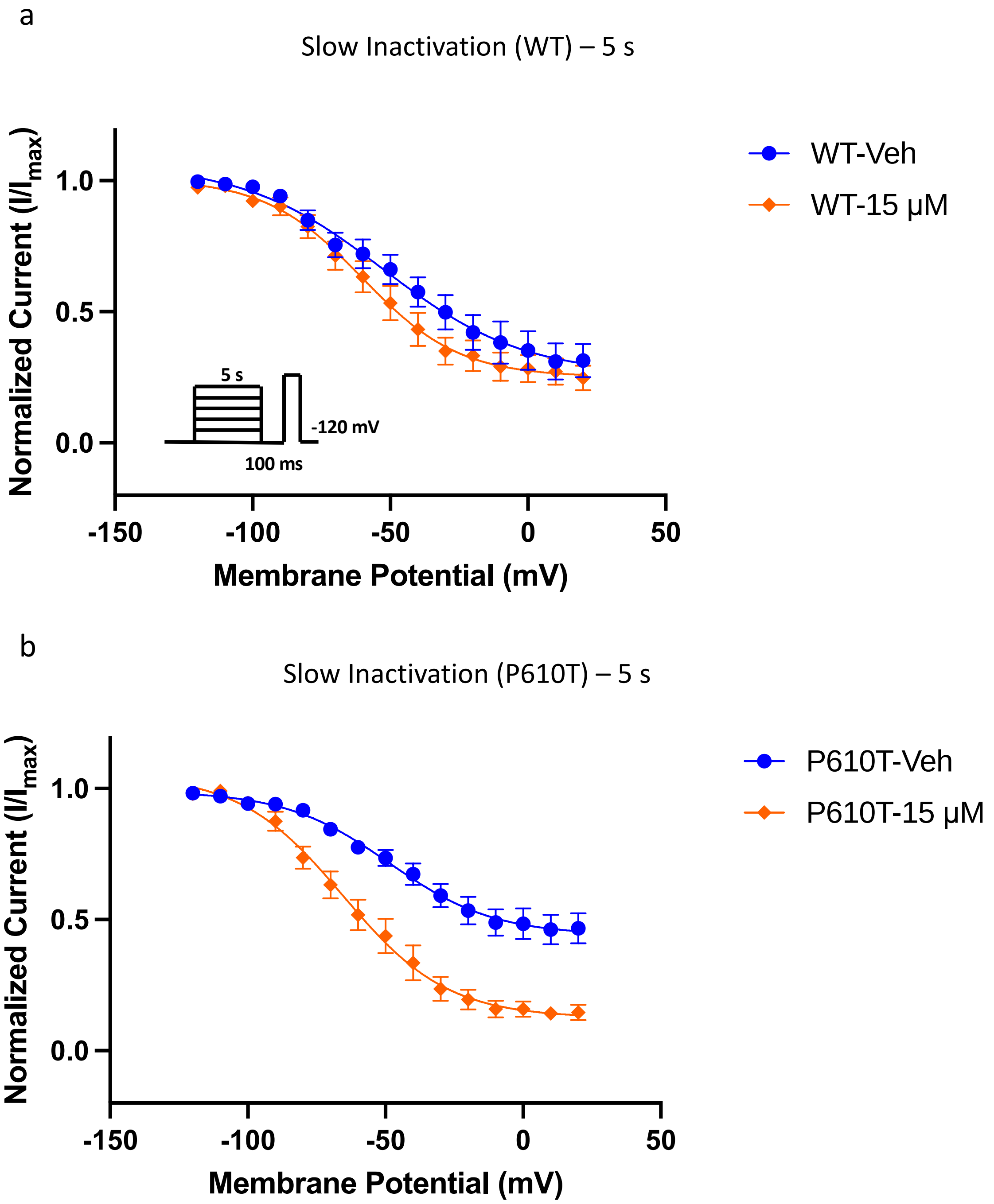

Figure S1 – CBN hyperpolarizes slow-inactivation curve in Nav1.7-P610T.

(**a-b**) Shows the curves associated with mutant and WT channels, transiently transfected into HEK cells. The time course that was used was 5 s. The concentration of CBN uses was 15 µM. Data shown as means ± SEM (n = 5-9).

a

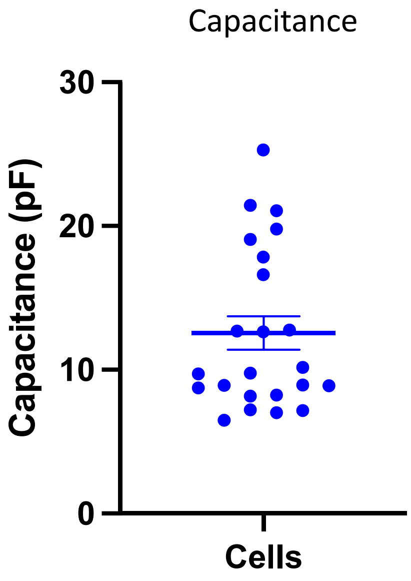

b

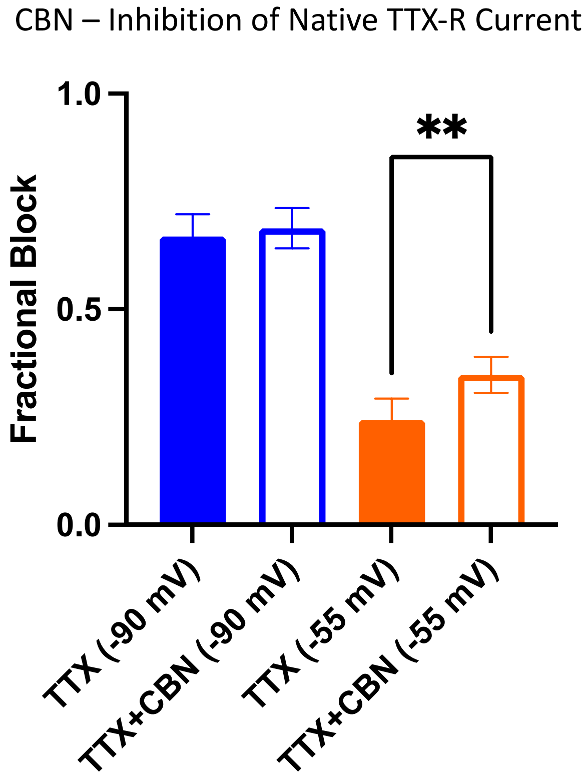

Figure S2 – CBN inhibits TTX-R current in freshly isolated DRG neurons.

(**a**) Shows the capacitance distribution of the cells that were tested at 10 μM. (**b**) Shows the fractional inhibition that CBN imparted at -90 and -55 mV. Data shown as means ± SEM (n = 21-26).
